# Are changes in depressive symptoms, general health and residential area socio-economic status associated with trajectories of waist circumference and body mass index?

**DOI:** 10.1371/journal.pone.0227029

**Published:** 2020-01-08

**Authors:** Theo Niyonsenga, Suzanne J. Carroll, Neil T. Coffee, Anne W. Taylor, Mark Daniel

**Affiliations:** 1 Health Research Institute, Faculty of Health, University of Canberra, Canberra, Australia; 2 School of Architecture and Built Environment, Healthy Cities Research Group, The University of Adelaide, South Australia, Australia; 3 Discipline of Medicine, The University of Adelaide, South Australia, Australia; 4 Department of Medicine, St Vincent’s Hospital, The University of Melbourne, Fitzroy, Australia; Ben-Gurion University of the Negev Faculty of Health Sciences, ISRAEL

## Abstract

**Objective:**

This study sought to assess whether changes in depressive symptoms, general health, and area-level socio-economic status (SES) were associated to changes over time in waist circumference and body mass index (BMI).

**Methods:**

A total of 2871 adults (18 years or older), living in Adelaide (South Australia), were observed across three waves of data collection spanning ten years, with clinical measures of waist circumference, height and weight. Participants completed the Centre for Epidemiologic Studies Depression (CES-D) and Short Form 36 health questionnaires (SF-36 general health domain). An area-level SES measure, relative location factor, was derived from hedonic regression models using residential property features but blind to location. Growth curve models with latent variables were fitted to data.

**Results:**

Waist circumference, BMI and depressive symptoms increased over time. General health and relative location factor decreased. Worsening general health and depressive symptoms predicted worsening waist circumference and BMI trajectories in covariate-adjusted models. Diminishing relative location factor was negatively associated with waist circumference and BMI trajectories in unadjusted models only.

**Conclusions:**

Worsening depressive symptoms and general health predict increasing adiposity and suggest the development of unhealthful adiposity might be prevented by attention to negative changes in mental health and overall general health.

## Introduction

Excess waist circumference and body mass index (BMI) are associated with the development of chronic diseases including cardiovascular diseases and type 2 diabetes [1]. The prevalence of overweight (BMI ≥ 25kg/m^2^) and obesity (BMI ≥ 30kg/m^2^) is rising worldwide [1, 2]. In Australia, the prevalence of overweight/obesity has been steadily increasing, from 56.3% in 1995 to 61.4% in 2007–08, 62.8% in 2011–12, and 63.4% in 2014–15 [3, 4]. Similarly, the prevalence of abdominal adiposity determined by waist circumference (men: ≥ 94cm; women: ≥ 80cm), rose from 45.0% in 1995 to 59.6% in 2007–08, then to 63.0% in 2011–12, slightly lessening to 62.1% in 2014/15 [3–5].

Individual-level sociodemographic characteristics, including socio-economic status (SES), are robustly linked to abdominal adiposity and overweight/obesity. Rising waist circumference and BMI are positively related to increasing age but inversely related to income and education, frequently used as individual SES measures, and subjective social status [6, 7]. Cross-sectional and longitudinal studies have documented inverse associations between obesity and individual SES measures, adjusting for demographic variables [8, 9]. In another longitudinal study, early-life family-level SES, measured as the family income-to-needs ratio at 1 month of age, subsequently predicted poor weight status in adolescence (elevated BMI at age 15 years) [10]. The relationship between early life SES and adolescent weight was mediated by maternal depressive symptoms [10].

Evidence of a link between mental ill health and obesity is important. Emotional and behavioural alterations are well established as related to multifaceted metabolic dysfunction [11]. Longitudinal studies have reported that greater depression or depressive symptoms increases the risk of developing obesity [12]. This relationship also runs the other way, with obesity linked to future developments of depression or depressive symptoms [12–14]. Some researchers have suggested that a ‘metabolic-mood syndrome’, defined as the state where alterations in the mood and the metabolism are clinically connected and mutually influencing each other [11], may exist. This link is arguably more than just the co-occurrence of mood symptoms and obesity [11, 13]. Longitudinal studies support a greater likelihood of depression leading to or causing obesity than obesity causing depression, with depression being a stronger risk factor for obesity than obesity for depression [12, 15].

Residential environment features, which define the context in which people live and the composition of people sharing a given context [16], contribute to health inequalities [17, 18]. Following the conceptual model of neighborhoods and health [19], studies have shown that residential environment features, such as the physical built environment and area-level SES, also play an important role in the development of obesity as physical and social environment features shape attitudes and health behaviors beyond individual-level characteristics [17].

Physical built environment refers to the human-made context in which human activity takes place. It includes features like healthy food access, public open space, community gardens, walkability and bikability, the presence of these resources shaping, to greater or lesser degrees, the health and wellness of local communities’ residents [20, 21]. Studies have shown that walkability, availability of public open space (POS) and healthful food outlets were all inversely related to weight status and obesity, while proximity to fast food was positively associated with weight status and obesity [9, 19, 22]. Studies demonstrated that local areas providing accessible built environmental factors conducive to physical activity (e.g., high walkability, mixed land use, retails areas for necessities of life) had consistently lower BMI [19, 22].

The social environment, in contrast to the physical built environment, is defined as the aggregated socio-economic features of areas expressing the composition of the residential area. It is most often expressed as area-level income and/or education, these being inversely related to unhealthful weight [23, 24]. Other measures of area-level SES have also been used, for example, the index of local area deprivation [25] and the index of relative social disadvantage [26], similarly found to be inversely related to rising body weight [27–29]. In Australian adults, greater increases in weight [5, 30] and waist girth have been observed for people in areas with greatest area-level socioeconomic disadvantage [5]. Recent studies have also included residential property values in the features of physical built environment to derive indices of both individual-level and area-level SES [31–34]. Studies showed that lower property values were linked to higher BMI and higher risk of obesity [8, 9, 34] as well as to higher cardiometabolic risks [31, 35].

Both physical built environments and social environments (proxied by area-level SES) are the key components of residential area features involved in this study. Given the links between physical built and social environmental features and both obesity and overweight beyond the individual-level characteristics [19, 36], it has been suggested that intervention strategies targeting obesity should include local area approaches [19, 22]. The merit of this proposition, however, requires assessment. Much of the research linking residential environment features to obesity is cross-sectional in design. More longitudinal studies are needed to provide strong evidence upon which to base intervention strategies. In addition, another limitation of much research to date is that analyses are adjusted only for a few *individual-level* covariates, notably demographic variables (e.g. age, sex, marital status, etc.) and SES indicators (e.g., education and income) [12].

Although adjustment for individual SES covariates is helpful, this strategy fails to account for the larger potentially confounding influence of the broader social environment as defined by the aggregated features of all individuals residing in a given area, not just the features of survey respondents or cohort participants. Correspondingly, as area-level SES covaries with physical built environmental features [37], analyses that account for the built environment are needed to estimate the independent effects of area-level SES, and factors shaped by area-level SES such as depression, on unhealthful weight.

Whilst some studies estimating environmental features on health do adjust analyses for built and social environmental features or feature such factors in complementary pathways [35, 38], this comprehensive approach to improving the estimation of the impact of environmental factors is not yet widely practiced. A thorough accounting is important because, although residential environment features such as area-level SES and individual psychosocial factors including mental disorders have been *separately* linked to abdominal adiposity and overweight/obesity, no study has yet estimated the *concurrent* impacts of residential environment features and individual-level psychosocial factors on waist circumference and BMI trajectories. Doing so could offer important insights as to the interrelationships shaping unhealthful body weight, because residing in a low SES area could indirectly shape unhealthful weight through a high incidence of depression that has additional impacts on weight, beyond the direct impact of a low SES area on healthful weight.

A particularly important consideration is how *changing* social and built environments relate to changes in outcomes and intermediate variables. Few studies have considered changes in residential environment features in relation to changes over time in weight trajectories. Of those that have, improvements in local-area food and physical activity environments were associated with reductions in BMI among persons who were obese or overweight at baseline after adjustment for individual-level covariates including duration of residence in the area, baseline BMI categories (normal, overweight, obese) [39]. Similarly, increases in the intensity of local-area development (e.g., higher density of walking destinations and population density) were associated with decreases in BMI and waist circumference after adjustment for individual-level covariates including self-rated health, cancer diagnosis and an indicator of moving during the follow-up period [36].

This study assessed the effects of baseline levels and rates of change in depressive symptoms, general health and area-level SES index derived from residential property values and operationalised as relative location factor, on trajectories of waist circumference and BMI, accounting for baseline covariates including individual-level socio-demographic factors, physical built and social environment indicators.

To our knowledge, no study has yet concurrently assessed whether *changes* in area-level SES and individual-level psychosocial factors predict changes in weight trajectories. Understanding these relationships has important implications for interventions to prevent overweight/obesity in urban planning and public health, as well as for clinical practice where shifting mental health and knowledge of the implications of patient locations could shape more effective, tailored weight treatment and maintenance strategies.

## Materials and methods

This study was part of the Place and Metabolic Syndrome (PAMS) project, which assessed the influence of local-area environments on individual-level cardiometabolic risks. The PAMS project utilised biomedical cohort data from the 10-year North West Adelaide Health Study (NWAHS) conducted in metropolitan Adelaide (South Australia) [40, 41]. The NWAHS cohort participants were observed across three waves with 4056 adult (aged 18 years or older, range: 18–90) participants at baseline (2000–3), n = 3205 (79% of the baseline sample) at Wave 2 (2005–06), and n = 2487 (61.3% of baseline) at Wave 3 (2008–10).

Not all NWAHS participants (n = 15) had addresses for which a geo-reference point could be assigned, with a resultant NWAHS baseline sample of n = 4041. The geocoded cohort sample was broadly representative of the Adelaide metropolitan population at that time, though older individuals were slightly over-represented and younger individuals under-represented [38]. As participants were nested within suburbs, the sample was restricted to participants who resided in suburbs with at least 5 participants (50 participants lost). The study was limited to participants living in metropolitan area at wave 1 (65 participants lost). Participants were excluded from the analytic sample if they moved their place of residence during the follow-up period between the first and second clinical visits (1185 participants lost). The analytic sample for this study comprised NWAHS participants with a geocoded residential address, living in an urban suburb with > = 5 participants and who had completed at least two of the three waves of data collection. The final analytic sample included 2871 participants, distributed within 126 suburbs, with 10.8% of the sample residing in the most disadvantaged areas (compared to 33.2% living in least disadvantaged areas).

Self-reported socio-demographic, psychosocial, and residential address information was collected via computer-assisted telephone interviews and paper questionnaires. Anthropometric data were measured at clinic visits at each wave. The participant residential address was used to create a geo-reference point enabling spatial joining with other datasets through a geographic information system.

Written informed consent was provided by all participants at each wave of data collection. Ethics approval for the PAMS Project was granted from three Human Research Ethics Committees: University of South Australia (P029-10 and P030-10); Central Northern Adelaide Health Service (Queen Elizabeth Hospital; Application No. 2010010); and South Australian Department for Health and Ageing (Protocol No. 354/03/2013). Additional information on the NWAHS cohort has previously been published elsewhere [38, 41].

### Measures

Outcomes of interest were trajectories of waist circumference and BMI, expressed using latent variables. Waist circumference, height and weight (for BMI as kg/m^2^) were measured by trained clinical staff during clinic visits. Waist circumference was measured to the nearest 0.1 cm using an inelastic tape maintained in a horizontal plane, with the subject standing comfortably with weight distributed evenly on both feet. Height was measured using a wall-mounted stadiometer (without shoes, to the nearest 0.5 cm). Weight was measured using standard electronic scales (light clothing without shoes, to the nearest 0.1 kg) [40, 41].

Independent variables were also trajectories (baseline and rate of change) of depression, general health and relative location factor, expressed as latent variables. Depressive symptomology was obtained using the Centre for Epidemiologic Studies Depression scale (CES-D) questionnaire, a validated screening test for depression and depressive disorders among general populations. The CES-D includes 20 questions where each item is rated by the participant on a 4-point Likert scale (coded from 0 to 3) with answers ranging from “Rarely or none of the time” to “Most or all the time”. Each item describes how the participant felt or behaved during the past week; for example: “I was bothered by things that usually don’t bother me” and “I talked less than usual”. Scores can range from 0 to 60, with higher scores indicating more depressive symptoms [42]. The CES-D has been used in many studies and has demonstrated moderate to high levels of validity and reliability [42, 43]. Depressive symptomology was only collected at waves 2 and 3, not at baseline.

General health measure was collected and expressed using the general health domain of the short form (SF) health status questionnaire (SF-36) [44]. The general health domain assesses the individual’s general health perceptions using 5 item contents. The first item is a subjective rating of health, worded as “Is your health: excellent, very good, good, fair, poor?”. The other four items express levels of agreement on each of the following content statements: “Seem to get sick a little easier than other people” (Sick easier); “As health as anybody I know” (As healthy); “Expect my health to get worse” (Health to get worse); “Health is excellent” (Health excellent) [45, 46]. The SF-36 general health domain was scored using the original 0–100 scoring algorithms based on summated ratings [44]. Studies of the SF-36 general health domain have yielded content, concurrent, criterion, construct, predictive evidence of validity and test-retest reliability [44, 47] with levels of internal consistence between 0.59–0.79, and estimates of reliability about 0.84 for the general health domain.

The relative location factor, an area-level SES index, was calculated based on individual residential property values. It is derived from a hedonic regression model built using residential property value sales transactions and selected residential property features, but blind to location, and expressed as the ratio of the actual price to the predicted price from the regression model [31]. The relative location factor has previously been used as an objective measure of local area (as opposed to individual) SES that emphasised the relative value of the location (where you live) rather than the residential property (what you live in) [32, 35].

A more traditional and commonly used expression of area-level SES, the index of relative socioeconomic disadvantage (IRSD), was also included as a covariate within analytic models. The IRSD, one of the socioeconomic indexes for areas, is a composite measure of deprivation constructed by the Australian Bureau of Statistics and derived from the 2001 Census [26].

Built environment measures used as covariates within analytic models, included: walkability; active public open space with physical activity resources (e.g., tennis courts); and a retail food environment index. These measures were operationalised within buffers constructed by radiating 1.6 km (approximately 1 mile) along the road network from each cohort participant’s residential address [48, 49]. This distance represents what is covered by an average adult walking for approximately 20 minutes [50].

The walkability index was constructed as the sum of deciles for dwelling density, road network density, and land use mix [51]. Availability of active public open space was defined as the count of active public open spaces (# parks) [52]. The retail food environment index, indicating the relative ‘unhealthfulness’ of the food environment, was expressed as the ratio of unhealthful food stores to healthful food stores, where unhealthful stores included major fast-food franchises and independent fast-food take-away stores, bakeries, sweet food retailers and convenience stores. Healthful stores included greengrocers, butchers, supermarkets (with > 200 m^2^ floor space), relatively healthful take-away options and health food shops [49].

### Statistical methods

Growth curve models using latent variables and a structural equations modelling (SEM) approach [53, 54] estimated the magnitude of relationships between the trajectories of the outcome measures (waist circumference or BMI), and changes in predictor variables (depression, general health and relative location factor). Models were adjusted for age, sex, education, income, smoking status, marital status, area-level SES (traditional expression) and built environment factors. The two latent variables, expressing baseline average (intercept) and rate of change over time (slope), were constructed for each outcome measure (waist circumference and BMI), and their key predictors (depression, general health, and relative location factor). Given three data waves over ten years, only linear growth curves were considered [54]. Unequally spaced times of measurements were used to define the trajectories rather than waves of assessment [55, 56]. The estimation process of models’ parameters within the structural equations approach relied on the full information maximum likelihood (FIML) approach that handles missing data and unequal time points, under the assumption that data are missing completely at random or at least at random with full models including at the individual level age, sex, education, income, smoking status as covariates or auxiliary variables [56, 57]. FIML estimation requires as well that observed outcome variables be derived from a multivariate normal distribution [55, 58].

Initial models estimated unadjusted associations between outcomes (waist circumference or BMI) and predictors (depression, general health, or relative location factor), including only one outcome measure and one main predictor at a time. For example, associations between the latent variables for relative location factor and waist circumference were modelled such that the intercept latent variable for relative location factor (i.e., baseline relative location factor) predicted intercept and slope for waist circumference, and the slope for relative location factor (i.e., change in relative location factor) predicted the slope for waist circumference. These models explored the directionality of associations between BMI, waist circumference, depression and general health (e.g., depression and general health predicting baseline and change in BMI and waist circumference, and conversely, BMI and waist circumference predicting baseline and change in depression and general health). The focus of the study remained, however, the direction from depression or general health to BMI or waist circumference.

**Fig 1:**
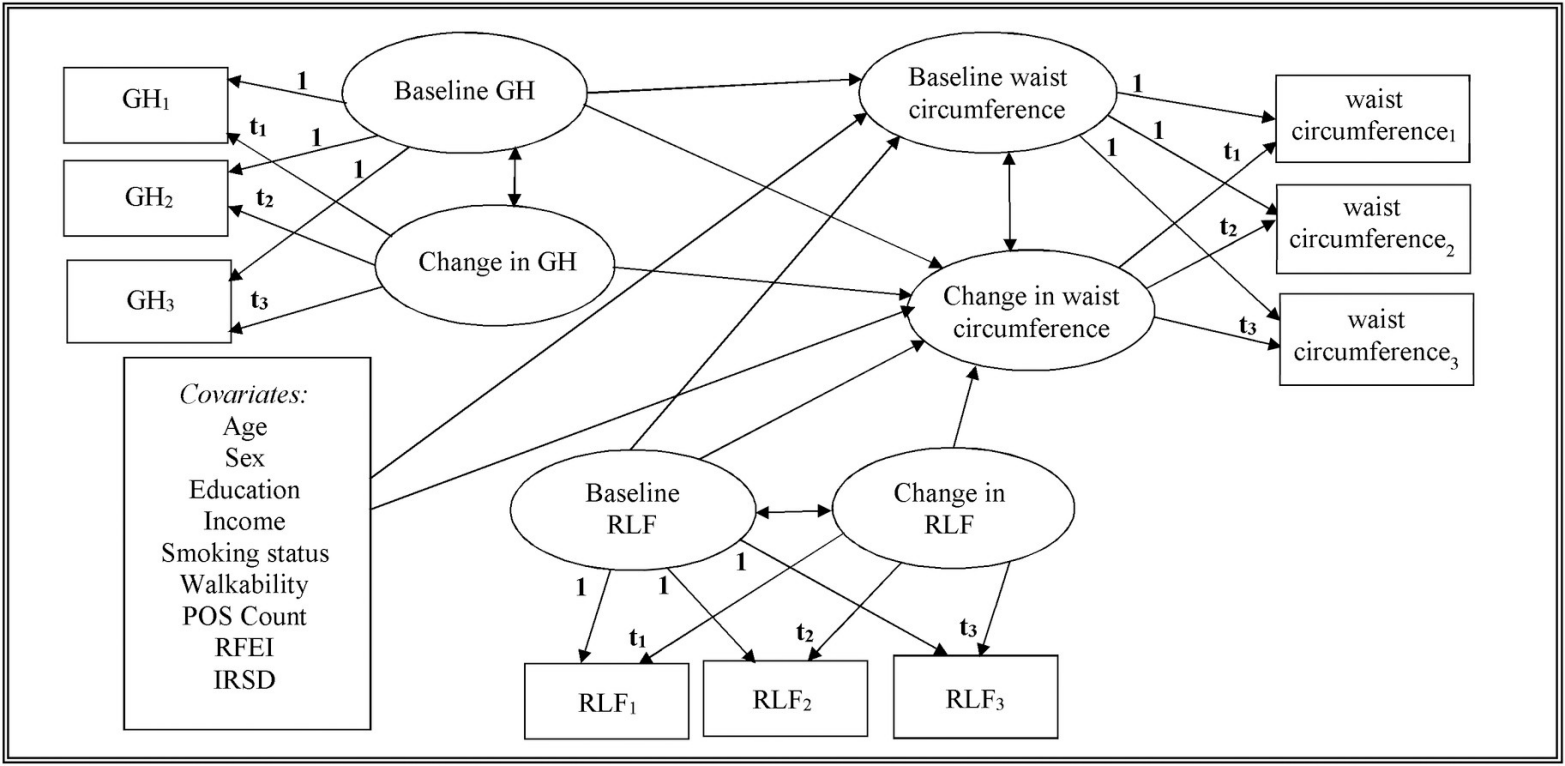
Full path diagram of the growth curve model for waist circumference. This path diagram illustrates the conceptual model of waist circumference trajectories as predicted by general health (GH) and Relative Location Factor (RLF) trajectories adjusting for various covariates. Subscripts 1 to 3 indicate measurements taken at waves 1 to 3; **t**_**1**_ to **t**_**3**_ represent unequal observation times; GH: General Health; RLF: Relative Location Factor; IRSD: Index of Relative Socioeconomic Disadvantage; POS: Public Open Space (# active parks); RFEI: Retail Food Environment Index. A path diagram model for predicting BMI trajectories can be done similarly.

Separate models using either depression or general health as main predictors were then fitted for each outcome variable (waist circumference and BMI). These models included: relative location factor latent variables, built environment indicators, index of relative socioeconomic disadvantage (area-level SES), and individual-level age, sex, education, income, smoking status as covariates. An example of a full path diagram of the modelled relationships includes both baseline status and change in waist circumference as outcomes, and baseline status and change in both general health and relative location factor as predictors (Fig 1). Analyses were performed using Mplus version 8 [53]. Statistical significance was set at 5% alpha.

## Results

Compared to the whole cohort at baseline, the analytic sample was on average 1.8 years older (significant difference with p<0.0001), but differed slightly and non-significantly in proportions for: education (1.8% more low education); marital status (2.9% more married or in de facto union); income (0.1% more in low income category); sex (0.9% less males); and smoking status (1.8% less smokers). The average follow-up was 10.4 years (minimum = 9.5, maximum = 11.6, and median = 10.5 years). Summary statistics (Table 1) show changes in sample mean values for variables measured over time (waist circumference, BMI, depression, general health, and relative location factor).

**Table 1:** Descriptive statistics of the analytic sample and associated area-level factors.

Variables	Sample size	Mean or proportion	Standard Deviation
***Individual-level characteristics (baseline unless otherwise stated)***			
Age (years)	2871	52.19	15.54
Sex (male: %)	2871	46.88	-
Education (Low: %)	2871	48.55	-
Income (Low: %)	2812	31.47	-
Smoking status (Yes: %)	2827	17.01	-
Married or De facto (Yes: %)	2861	63.98	-
Waist circumference (cm) Wave 1	2869	91.91	14.46
Waist circumference (cm) Wave 2	2593	94.79	14.42
Waist circumference (cm) Wave 3	1978	95.26	14.60
BMI (kg/m^2^) Wave 1	2871	27.98	5.38
BMI (kg/m^2^) Wave 2	2613	28.22	5.48
BMI (kg/m^2^) Wave 3	1979	28.70	5.36
Depression (CES-D) Wave 2	2814	6.34	8.28
Depression (CES-D) Wave 3	2041	8.76	9.13
General Health Wave 1	2861	68.00	20.09
General Health Wave 2	2638	67.99	19.98
General Health Wave 3	2083	66.87	19.52
***Environmental features***			
Relative Location Factor Wave 1	2815	5.57	2.89
Relative Location Factor Wave 2	2804	5.46	2.90
Relative Location Factor Wave 3	2140	5.50	2.89
Retail Food Environment Index	2871	2.26	1.53
Active Public Open Space Count	2871	5.77	2.96
Walkability index	2865	16.34	5.91
IRSD (Area-level SES)	2871	956.94	104.29

Notes

BMI: Body Mass Index; CES-D: Centre for Epidemiologic Studies Depression scale; IRSD: Index of Relative Socioeconomic Disadvantage; SES: Socio-Economic Status.

These changes were confirmed by estimated parameters (latent variable slopes) of the growth curves (Table 2). Waist circumference, BMI and depression each increased over time (waist circumference: 0.340 cm per year [95%CI: 0.295, 0.385]; BMI: 0.090 kg/m^2^ per year [95%CI: 0.074, 0.106]; depression: 0.555 points per year [95%CI: 0.451, 0.659]), while general health and relative location factor decreased (general health: -0.282 points per year [95%CI: -0.360, -0.204]; LVF: -0.019 points per year [95%CI: -0.043, 0.005]) (the decrease for relative location factor was not statistically significant).

**Table 2:** Trajectories of waist circumference, body mass index, depression, general health and relative location factor: estimated intercept (I) and slope (S) coefficients and standard errors (SE).

***Variables***	***Estimated Trajectories*: *intercept [I (SE)] and slope [S (SE)]***	***Pairwise associations between trajectories*: *Beta-coefficients for intercepts and slopes (SE)***	
		***Waist circumference***	***Body mass index***
Depression	n = 2490 I = 2.904 (0.394) **** S = 0.555 (0.053) ****	β_II_ = 0.932 (1.959) β_SI_ = 0.054 (0.029) β_SS_ = 0.115 (0.038) ***	β_II_ = 0.295 (0.154) β_SI_ = 0.025 (0.012) ** β_SS_ = 0.037 (0.012) ***
General Health	n = 2713 I = 69.108 (0.483) **** S = -0.282 (0.040) ****	β_II_ = -0.234 (0.020) **** β_SI_ = -0.007 (0.001) **** β_SS_ = -0.650 (0.040) ****	β_II_ = -0.071 (0.008) **** β_SI_ = -0.001 (0.001) β_SS_ = -0.208 (0.053) ****
Relative Location Factor	n = 2373 I = 5.676 (0.320) **** S = -0.019 (0.012)	β_II_ = -0.583 (0.115) **** β_SI_ = -0.022 (0.008) *** β_SS_ = -9.575 (0.954) ****	β_II_ = -0.217 (0.041) **** β_SI_ = -0.007 (0.003) *** β_SS_ = -6.130 (0.624) ****
***Variables***		***Pairwise associations between trajectories*: *Beta-coefficients for intercepts and slopes (SE)***	
		***Depression***	***General Health***
Waist Circumference (cm)	n = 2869 I = 92.179 (0.324) **** S = 0.340 (0.023) ****	β_II_ = 0.015 (0.013) β_SI_ = 0.002 (0.002) β_SS_ = 0.278 (0.103) ***	β_II_ = -0.358 (0.028) **** β_SI_ = -0.023 (0.002) **** β_SS_ = -1.032 (0.111) ****
Body Mass Index (kg/m^2^)	n = 2871 I = 27.731 (0.125) **** S = 0.090 (0.008) ****	β_II_ = 0.110 (0.035) *** β_SI_ = 0.012 (0.005) ** β_SS_ = 0.539 (0.183) ***	β_II_ = -0.767 (0.077) **** β_SI_ = -0.046 (0.006) **** β_SS_ = -0.855 (0.294) ***

Notes

P-values

****: p<0.001

***: p<0.01

**: p<0.05

*: p<0.1

I: Intercept; S: Slope; SE: Standard Error; β_I_: regression coefficient associated to intercept; β_S_: regression coefficient associated to slope.

Unadjusted relationships between waist circumference, BMI, depression, general health and relative location factor trajectories are given in Table 2 (simple regression coefficients resulting from intercept regressed on intercept (β_II_); slope regressed on intercept (β_SI_); slope regressed on slope (β_SS_); standard error (SE)). Increasing (worsening) depression was associated with increases in both waist circumference and BMI (β_SS_-coefficients: 0.115 [95%CI: 0.041, 0.189] and 0.037 [95%CI: 0.013, 0.061], respectively). Changes in general health and relative location factor were both inversely associated with change in waist circumference (β_SS_-coefficient for general health: -0.650 [95%CI: -0.728, -0.572]; β_SS_-coefficient for relative location factor: -9.575 [95%CI: -11.445, -7.705]) and change in BMI (β_SS_-coefficient for general health: -0.208 [95%CI: -0.312, -0.104]; β_SS_-coefficient for relative location factor: -6.130 [95%CI: -7.353, -4.907]. That is, worsening general health (i.e., decreasing over time) was associated with increasing waist circumference and BMI. Similarly, worsening relative location factor was associated with increasing waist circumference and BMI.

Although not the focus of the study, the potential bidirectionality of associations suggested by the literature was supported by the results provided in Table 2. Results showed that BMI does predict significantly both depression and general health trajectories. Models with waist circumference as a predictor were statistically significant for general health. In addition, change in waist circumference did predict significantly significant changes in depression.

Results of the fully adjusted growth curve models (i.e., adjusted for individual-level sociodemographic, area-level SES (traditional expression) and residential built environment features) are presented in Table 3. For the outcomes of interest, baseline general health was inversely and statistically significantly associated with baselines of waist circumference (β-coefficient: -0.163 [95%CI: -0.198, -0.128]) and BMI (β-coefficient: -0.059 [95%CI: -0.075, -0.043]). Baseline relative location factor was also inversely related to baseline waist circumference and baseline BMI, though associations were not statistically significant.

**Table 3:** Growth curve models predicting baseline and change in waist circumference and body mass index.

	**Outcome: Waist Circumference**		**Outcome: Body Mass Index**	
	Intercept (SE)	Change (SE)	Intercept (SE)	Change (SE)
Depression (n = 2386)				
Depression^1^: Intercept	0.101 (0.098)	0.010 (0.007)	0.045 (0.054)	0.003 (0.003)
Change	-	0.087 (0.033) ***	-	0.022 (0.011) **
RLF^1^: Intercept	-0.234 (0.143)	-0.017 (0.019)	-0.064 (0.061)	-0.003 (0.005)
Change	-	-2.172 (1.838)	-	-0.422 (0.445)
Age	2.603 (0.312) ****	-0.135 (0.026) ****	0.425 (0.111) ****	-0.085 (0.008) ****
Sex (male)	12.543 (0.588) ****	-0.086 (0.046) *	0.533 (0.251) **	-0.022 (0.014)
Education^2^: Low	1.498 (0.533) ***	-0.025 (0.040)	0.558 (0.227) **	0.004 (0.013)
Income^2^: Low	0.461 (0.751)	0.014 (0.055)	0.143 (0.307)	-0.010 (0.020)
Smoking (Yes)	-2.666 (0.820) ***	0.284 (0.064) ****	-1.184 (0.347) ***	0.065 (0.024) ***
Married/De facto (Yes)	0.430 (0.594)	-0.045 (0.055)	0.351 (0.226)	-0.022 (0.016)
Walkability	0.316 (0.302)	-0.008 (0.026)	0.048 (0.119)	-0.006 (0.009)
RFEI	-0.076 (0.270)	0.022 (0.022)	-0.135 (0.125)	0.009 (0.007)
POS Count	-0.271 (0.357)	0.007 (0.024)	-0.045 (0.125)	-0.003 (0.007)
IRSD (Area-level SES)	-1.153 (0.410) ***	0.003 (0.030)	-0.542 (0.166) ***	-0.002 (0.010)
	AIC = 102734.709		AIC = 88815.722	
GH (n = 2394)				
GH^1^: Intercept	-0.163 (0.018) ****	-0.001 (0.001)	-0.059 (0.008) ****	<0.001 (<0.001)
Change	-	-0.193 (0.052) ***	-	-0.046 (0.017) ***
RLF^1^: Intercept	-0.206 (0.137)	-0.019 (0.015)	-0.055 (0.059)	-0.004 (0.005)
Change	-	-1.876 (1.256)	-	-0.457 (0.400)
Age	2.366 (0.298) ****	-0.155 (0.027) ****	0.339 (0.110) ***	-0.089 (0.009) ****
Sex	11.876 (0.570) ****	-0.095 (0.046) **	0.291 (0.248)	-0.024 (0.014) *
Education: Low	1.114 (0.517) **	-0.014 (0.042)	0.411 (0.221) *	0.005 (0.015)
Income: Low	-0.322 (0.745)	0.032 (0.056)	-0.127 (0.303)	-0.005 (0.020)
Smoking	-3.653 (0.817) ****	0.290 (0.061) ****	-1.530 (0.350) ***	0.068 (0.024) ***
Married/De facto (Yes)	0.505 (0.588)	-0.048 (0.055)	0.375 (0.227) *	-0.023 (0.016)
Walkability	0.254 (0.292)	-0.006 (0.026)	0.026 (0.115)	-0.005 (0.009)
RFEI	-0.169 (0.276)	0.021 (0.021)	-0.167 (0.127)	0.009 (0.007)
POS Count	-0.284 (0.342)	0.008 (0.023)	-0.048 (0.118)	-0.003 (0.007)
IRSD (Area-level SES)	-0.966 (0.401) **	0.003 (0.029)	-0.479 (0.163) ***	-0.003 (0.010)
	AIC = 135112.174		AIC = 121210.022	

Notes

P-values identified

****: p<0.001

***: p<0.01

**: p<0.05

*: p<0.10

: p>0.10

^1^latent variables with intercept (baseline) and slope (rate of change over time)

^2^Reference category “high” education; CES-D: Centre for epidemiologic studies depression scale; SF36GH: Short Form (36) general quality of health scale; RLF: Relative Location Factor (proxy of area-level SES measure); Walkability: Walkability index; RFEI: Retail food environment index; POS Count: Count of active public open spaces (# parks); IRSD: Index of Relative Socioeconomic Disadvantage (Australian Bureau of Statistics Socio-Economic Indexes For Areas measure); AIC: Akaike Information Criterion.

Regarding longitudinal relationships in the fully adjusted models, baseline values for depression, general health and the relative location factor were not significantly associated with changes in waist circumference or BMI. However, increasing depression and worsening general health were each statistically significantly associated with increases in waist circumference (depression, β-coefficient: 0.087 [95%CI: 0.023, 0.151]; general health, β-coefficient: -0.193 [95%CI: -0.294, -0.092]) and BMI (depression, β-coefficient: 0.022 [95%CI: 0.001, 0.043]; general health, β-coefficient: -0.046 [95%CI: -0.079, -0.013]). The decrease in the relative location factor values was not significantly associated with changes in waist circumference or in BMI.

Among area and individual level covariates: index of relative socioeconomic disadvantage was statistically significantly inversely associated with baseline values of waist circumference and BMI trajectories; age was directly associated with baseline waist circumference and BMI but inversely related to change in the outcomes over time; similarly, sex (male relative to female) was directly related to baseline waist circumference but inversely associated with change in waist circumference, though at borderline significance (p<0.10) in the waist circumference model including depression; sex was directly associated with baseline BMI only in models including depression, and inversely associated with change in outcomes over time, at p<0.10 for the model involving general health; education (low compared to high) was directly related to baseline waist circumference and BMI, at p<0.10 in the BMI model including general health; and lastly, being a smoker was associated with baseline outcome measures (inverse associations) and change in outcomes over time (positive associations).

Fully adjusted waist circumference and BMI latent growth models were more parsimonious (smaller AIC values), compared to corresponding intercept-only models in which only the means (intercept and covariates), variances (intercept and covariates) and residual variances are free parameters. Conventional fit indices for SEM (e.g., χ^2^, CFI and TLI, RMSEA, SRMR) [59, 60] have not been reported here since they are not available for complex growth curve models fitted to time-unbalanced longitudinal data using the structural equation modelling (SEM) approach [61].

## Discussion

This study assessed the independent associations between baseline status and change in depression, general health and relative location factor, and change in waist circumference and BMI, accounting for individual and area level covariates. For our population of predominantly middle-aged adults in Adelaide, we conclude that 1) worsening depressive symptomology is related to increases in waist circumference and BMI, 2) worse baseline general health is associated with greater waist circumference but not BMI at baseline, and worsening general health is related to increases in waist circumference and BMI, and 3) neither baseline status nor change in a resident-specific expression of area-level SES were statistically related to baseline status and change in either waist circumference or BMI, whilst a traditional measure of area-level SES, expressed only at baseline, was inversely related to baseline waist circumference and BMI but not to changes in these measures over time.

Our findings on associations between depression and anthropometric measures of adiposity align with the results from previous work in this area [12, 13, 62, 63]. Miller et al. [62] in their pathway models found that the primary causal pathway was from depression to increased adiposity (possibly through physical inactivity) and to an increase in inflammatory markers. On the other hand, Luppino et al. [12] in their meta-analysis of longitudinal studies highlighted a bidirectional association between depression and obesity (especially abdominal adiposity) in which prior obesity increased the risk for depression and depression increased the likelihood of subsequent obesity. In univariate analyses, our results indicated bidirectional associations. Our results, and those of Shelton and Miller [63] and Luppino et al. [12], all indicate that further longitudinal research is needed to elucidate the temporal pathways underlying depression-obesity associations, including both mediating and moderating factors such as alcohol and tobacco consumptions and dietary patterns.

Significant inverse associations between health-related quality of life and BMI have similarly been highlighted elsewhere. Cameron et al. indicated a bi-directional association, although in their study, baseline scores of one variable were used to predict changes over time in scores of the other variable and vice versa [64]. In other studies, waist circumference exhibited strong inverse associations with SF-36 dimensions including general health, but these associations were either cross-sectional and/or limited to specific sex and age groups [65].

Our findings of negative effects of both area-level SES measures (relative location factor and index of relative socioeconomic disadvantage) on BMI and waist circumference at baseline (inverse relationships or associations), although non-significant for relative location factor in adjusted models, are to some extent consistent with previous literature [9, 27–29, 66].

Both relative location factor measures (baseline and rate of change), were significantly inversely associated with both BMI and waist circumference trajectories in unadjusted models, but the associations became non-significant in adjusted models. These findings align with what other studies reported. In two cross-sectional studies, using residential property values, Drewnowski et al. found an inverse association with BMI values among women [67], and with obesity risk in Seattle and Paris [8]. In longitudinal studies, authors reported strong and inverse associations between baseline residential property values and the baseline obesity prevalence [9, 68], but no significant association for either one-year weight change [9], or with one-year change in BMI values [68].

In our study, the index of relative socioeconomic disadvantage, commonly used measure of area-level SES in Australia, had significant negative associations with baseline values but not rates of change for both BMI and waist circumference (non-significant inverse relationships). This is inconsistent with some study findings reported for BMI and for central adiposity. Indeed, Berry et al. [27] found that area-level SES was inversely related to change in BMI, while Kwarteng et al. [66] reported that local high poverty areas were more likely to reflect increases in central adiposity rates over time, after adjustment for individual covariates. Furthermore, Coogan et al. [29] reported an inverse relationship between area-level SES and weight gain among women, and Powell-Wiley et al. [28] confirmed this relationship for both genders. They found that the association between weight change and area-level deprivation was modified by length of residence in the neighborhood location [28].

Other studies reported findings consistent with ours, although based on the prevalence of obesity and weight gain. Indeed, Drewnowski et al. [9, 68] reported that traditional area-level SES had no significant impact on the short-term 12-month weight change but a strong and inverse association with baseline obesity prevalence. They speculated that weight trajectories may be driven by individual and behavioural factors rather than local area SES [68]. This aligns with our findings where individual depression symptoms and general health status appear significantly more important than both the relative location factor and the index of relative socioeconomic disadvantage.

Features of physical and social environments have been linked to general health and mental health (especially depressive symptoms) which are, in turn, linked to overweight/obesity and abdominal adiposity. Therefore, studies examining residential environment features, in the context of both BMI and waist circumference changes, should be considered within the same conceptual framework of neighborhoods and health, looking at both individual-level and area-level features simultaneously. Reported studies are limited by the absence of models which account for various pathways involved in the BMI/waist circumference and depressive symptomatology/general health/area-level SES relationships, including the mediation and moderation processes. Such models would help to understand the proximal and distal impacts of both depression and general health (proximal) as well as residential environment features (distal) on waist circumference and BMI, after adjustment for individual covariates including socio-demographics.

A key distinction of our analysis is that, accounting for other influences, we found no significant associations between any physical built environment feature and baseline or change in waist circumference or BMI. This was unexpected but is well supported by our data and models, and is not untenable given the design limitations of much previous work, for example, cross-sectional studies that have reported inverse associations between BMI and count of parks or proportion of land covered by park space [69], and positive associations between density of neighborhood fast food outlets and the risk of obesity among older adults [70]. Such studies have a high risk of confounding and lack any basis for clarifying what comes first (temporal antecedence). Far fewer longitudinal studies have examined these associations over time [36, 71, 72]. Prospective studies that exist have reported 1) positive associations between increasing weight and waist circumference and density of fast-food outlets, but inverse associations between walkability and weight and waist circumference [71] and 2) inverse associations between BMI and waist circumference, and residential environment features including walkability, density of walking destinations, population density and lower percent residential area [36]. In one prospective study, however, the investigators noted that individual walkability components, such as residential density, connectivity, and land use mix, were associated with walking behaviours not obesity [72]. Few studies have included multiple built environment factors concurrently within models, particularly when including individual psychosocial factors. Our finding of no significant link between built environment factors and adiposity reflects such measures correlating with area-level SES, and thus confounding of the relationship between built environment, BMI and waist circumference [35].

Our study did not aim to identify mediation mechanisms or moderation effects. Potential moderators (e.g., age, sex, social support, environmental factors) could have been tested but we strove to focus on main effects only, not sub-group analyses. This reflects small group sizes and inadequate statistical power for evaluation. Further mediation and/or moderation analyses will be needed to test the effects of neighbourhood factors including the food environment (e.g., fast food outlets) and public open spaces (for physical activity) on waist circumference and BMI, and the modulating effects of psychological factors. It is important also to investigate group trajectories to determine specific patterns of change (in BMI and waist circumference) and estimate the effects of both depression and general health within devised group trajectories.

Strengths of this study include the prospective nature of the data collected and the length of follow-up period, enabling examination of baseline status and change in predictors and outcomes. The use of objective measures is another strength with clinically assessed anthropometric variables and built environment indicators from geographic information systems. Depressive symptomology and general health on the other hand were not measured clinically, but via self-reported responses to questionnaires. While reliable and valid, such measures may bear more uncertainty which would bias model findings to the null. Therefore, relationships reported here between depression and general health in relation to BMI/waist circumference are likely under-reported.

The first key limitation of our study is the use of baseline measures for built environment characteristics which do not express how long individuals were exposed to environmental features, or whether the environment changed over the follow-up period. The second key limitation is relative to models’ assumptions. For models’ parameter estimation, we first assumed that both measurement and structural models are well defined. Second, with respect to the measurement models, as frequently done, we assumed that error terms are uncorrelated across waves and error variances are equal over time. Other limitations include the possibility of residual confounding given the omission from models of relevant unmeasured influences at both individual and neighborhood levels (e.g., individual physical activity and eating behaviors, medications such as anti-depressants, other area-level factors including safety or cleanness, and public transportation. Finally, the issue of missing data, particularly the attrition across study waves, may have important effects on our findings, although the study attempted to minimize these effects at the analysis stage. Indeed, to deal with missing data, we used the full information maximum likelihood estimation approach and the inclusion of auxiliary variables into the SEM [57, 73]. However, we are unable to completely control for potential bias due to the attrition, especially if this is due to self-selection [74, 75]. As other authors pointed out, it is possible that non-random selection in or out of residential areas accounts for some of the associations between residential environment features (e.g., area-level SES) and BMI and waist circumference [76, 77].

Our study builds on and expands the literature on obesity, depressive symptomology and general health. It highlights important directions of change over time in these measures, and estimates the magnitude of associations between adiposity, depression and general health while accounting for local area built and social environmental features. Our findings support the importance that both depression and general health play in the evolution of adiposity over time, suggesting that there may be potential benefits from the better management of individual depressive symptoms to reduce the risk of increasing adiposity than a focus on the built environment *per se*. We found little support for any impact of built environment on weight status but some support for the influence of area-level SES on adiposity.

The study findings have clinical implications given the inter-relationships between depression, perceived general health, waist circumference and BMI, and the impact these anthropometric measures of adiposity and overweight/obesity have as risk factors to chronic diseases such as type 2 diabetes and cardiovascular diseases. As Davillas et al. pointed out, addressing any of these problems in isolation would be ineffective [78]. There is a need of a multi-disciplinary management care and detailed clinical guidelines to help physicians prevent and treat both mental health and obesity while promoting high quality of general and mental health [78–80]. Moreover, the impairment in general and mental health, adiposity and overweight/obesity and the need of clinical guidelines are clearly important in the primary care settings as, in Australia, 75% of all medical consultations take place in general practice (GP)’s offices, and more than 85% of the population access a GP each year [81]. Using GP-based data, Niyonsenga et al. [82] pointed out high rates of co-occurrence of mental health and both asthma and COPD as other chronic conditions to be considered. This justifies the necessity of a multi-facet approach to overall management care to improve patients’ chronic conditions. This service delivery needs to start in primary care settings and/or be a result of a shift from acute to primary health care. Finally, as Fitzpatrick pointed out [83], the evidence-based guide for obesity treatment in primary care puts the primary care physicians in the centre of the framework to build and coordinate a multidisciplinary team that provides integrated care, and monitors the different aspects of patient’s life as a whole to maximize the patient’s successful health management [13, 83].

## Conclusions

Findings support the importance of depression and general health in the evolution of adiposity over time. Depression and low quality of general health appear to be more important to increasing adiposity than baseline measures of both built and social (area-level SES) environment features. It is plausible that the former (depression and general health) are more proximal at the individual level in the causal chain, therefore more likely to affect changes in waist circumference and BMI, whereas the latter (residential environment features) have a more distal impact broadly.
